# Heterotopic ossification following anti-NMDA receptor encephalitis: a case report

**DOI:** 10.1186/s12883-016-0747-4

**Published:** 2016-11-21

**Authors:** Dongmei Wang, Shengnan Wang, Xiaoxian Huang, Qun Wang

**Affiliations:** Department of Neurology, Nanfang Hospital, Southern Medical University, 1838 Northern Guangzhou Avenue, Guangzhou, 510515 Guangdong China

**Keywords:** Heterotopic ossification (HO), Anti-NMDA receptor encephalitis

## Abstract

**Background:**

Heterotopic ossification (HO) is defined as the formation of true bone tissue in non-osseous tissues. HO may occur under several conditions such as soft tissue injury, central nervous system injury and many other diseases like arthopathies, and vasculopathies. The underlying mechanisms of HO are not well elucidated. Anti-NMDA receptor encephalitis is a newly recognized autoimmune mediated disease which is predominant in young female patients with ovarian teratomas. Encephalitis complicated with HO has rarely been reported.

**Case presentation:**

Here we report a case of anti-NMDA receptor encephalitis with severe muscle ossifications. A 15 years old female patient presented with fever, changed mental status of confusion, rigidity of the arms and legs, and oral-facial dyskinesias. Diagnosis of anti-NMDA receptor encephalitis was confirmed by detection of anti-NMDA receptor antibodies both in serum and CSF. Due to the severity of the disease, 3-weeks’ intensive care and mechanical ventilation were administrated for the patient. Image of pelvic CT and MRI of the patient showed dynamic changing process of HO. The muscles showed edema and scattered inflammation at the very beginning, and then gradually formed mature bone tissue.

**Conclusions:**

Anti-NMDA receptor encephalitis often presents with severe neurologic symptoms and requires long time intensive care and mechanical ventilation, which makes the patient easily complicate with HO. More studies are required to elucidate the mechanisms of HO and more attention should be paid to patients with encephalitis who might develop severe muscle ossifications requiring early interventions.

## Background

Heterotopic ossification (HO) is a pathological process of mature, lamellar bone formation in non-osseous tissues (muscle and connective tissue) [1]. There are many causes of HO, including soft tissue injury, central nervous system (CNS) injury, and others like arthopathies, vasculopathies and inheritance [2]. The pathogenesis of HO is unclear. Recent work has shown that the inappropriate differentiation of mesenchymal cells into osteoblastic stem cells in response to unidentified factors such as pool of available calcium in adjacent skeleton, soft tissue edema, and vascular stasis, tissue hypoxia is thought to be involved in the process [2]. Neurogenic HO following traumatic brain injury (TBI) and spinal cord injury (SCI) has been widely reported. However, HO associated with encephalitis is rarely reported [3, 4].

Anti-N-methyl-d-aspartate (anti-NMDA) receptor encephalitis has recently been reported in young women with ovarian teratomas [5]. The clinical features usually evolve as follows: psychiatric presentations with seizures, conscious disturbance, dysautonomia and movement disorder and recovery after treatment [6].

No Anti-NMDA receptor encephalitis with HO has been reported. Herein we present a case of anti-NMDA receptor encephalitis with ossifications of multiple muscles.

## Case presentation

A 15-year-old previously healthy female was brought to our emergency department due to loss of consciousness and witnessed generalized seizures. On admission, her physical examination revealed low-grade fever of 38 °C, changed mental status of confusion, rigidity of the arms and legs, and oral-facial dyskinesias. Other nervous system examinations were not remarkable. Cranial magnetic resonance imaging (MRI) was normal, and cerebrospinal fluid (CSF) tests showed cells counts, levels of glucose and protein were all within normal ranges. Electroencephalograph (EEG) demonstrated diffused 2.5–3 Hz delta waves with amplitude of 10–40 μV. The patient was given anti-viral therapy for presumed viral encephalitis. One day later, she experienced another generalized seizure followed by unconsciousness, thus she was intubated and transferred to intensive care unit (ICU).

**Fig. 1:**
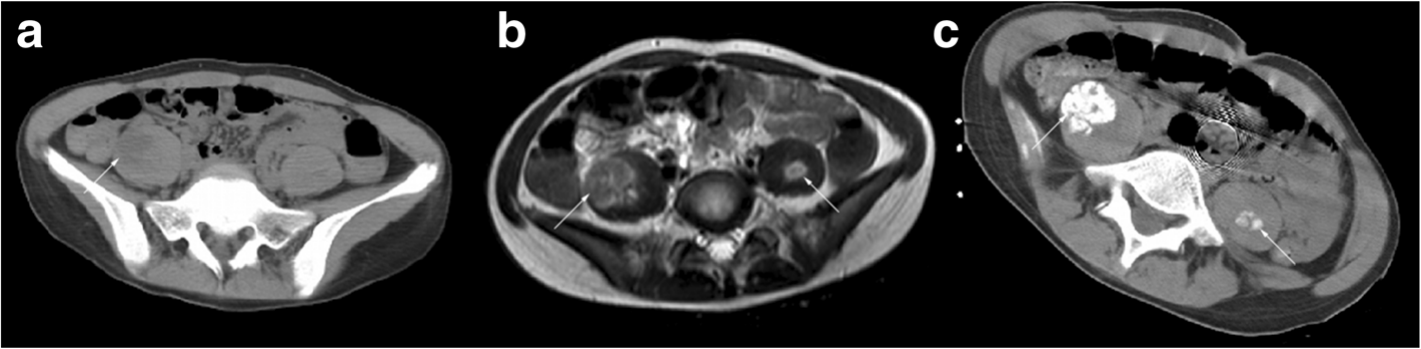
The pelvic CT and MRI of the patient. **a** The 4th day on admission, pelvic CT revealed hypointensity and edema of the right psoas major (*arrow*). **b** The 10th day on admission, T2 weighted MRI showed abnormal signals of the bilateral psoas major (*arrows*). **c** The 17th day on admission, pelvic CT showed diffused ossifications of the bilateral psoas major (*arrows*)

Blood test results were unremarkable except for decreased parathyroid hormone (PTH) of 10.40 pg/mL (normal range: 15–65 pg/mL) and serum calcium of 2.16 mmol/L (normal range: 2.20–2.65 mmol/L). Continuous EEG demonstrated non-convulsive status epileticus, requiring high dosage of anticonvulsants such as phenobarbital and general anesthesia. Cranial MRI was repeated and revealed enhancement of the pia matter. Anti-NMDA receptor antibodies were detected in both the serum and CSF. Thoracic, abdominal and pelvic computed tomography (CT) was performed for the patient and revealed no tumors. Pelvic CT revealed hopointensity and edema of the right posas major (Fig. 1) suggesting that a MRI scan should be ordered for the patient. Six days later, pelvic MRI with contrast was scanned which showed edema and slightly enhancement of the bilateral posas major (Fig. 1) and obturator internus implying muscles inflammation. CT guided needle biopsy of the right major posas was taken. As showed in Fig. 1, diffused ossifications of the bilateral posas major were found in CT scan. Scattered muscle inflammation but no ossification was observed in the biopsy specimen. The patient was treated by Intravenous immunoglobin (IVIg) (0.4 g/kg/d) for 5 days followed by plasma exchange. The patient’s neurologic status improved gradually. Physical therapy was recommended for the patient. Because of the financial problem, the patient was discharged. Even though, we trained her parents to do passive movements for the patient at home. Two months later, the patient walked to our clinic by herself. She had no complaint of back pain, joints pain or mobility problem. However, her neurologic examination revealed decreased orientation and intelligence.

## Conclusions

Brain injury and spinal cord injury are the main causes of neurogenic HO with the incidence of 10–23% and 40–50% respectively [7]. Although several HO cases after viral encephalitis have been reported [4], our case is the first report of autoimmune encephalitis with HO.

Anti-NMDA receptor encephalitis has recently become a topic of interest in medical community since the diagnosis method by antibodies detection in serum or CSF become available. Like other neurogenic HO, the mechanism of HO after anti-NMDA receptor encephalitis is not clear. Recent studies reveal that pathogenesis of HO involves three requisite components: including osteogenic precursor cells, inducing agents, and a permissive environment [8]. Risk factors for neurogenic HO including the severity of the neurologic injury, presence of the spastic as compared with flaccid paralysis, multiple injuries at the time of trauma. Prolonged coma and artificial ventilation are important risk factors. Increased duration of artificial ventilation (extremely common in patients with TBI and SCI) may alter the homeostasis of the patients, especially in terms of electrolytes and acid-base balance [8]. In anti-NMDA receptor encephalitis cases, a lot of patients require the support of artificial ventilation, which increases the risks of HO. Muscle spasticity is extremely common in CNS injured patients. The spasticity causes muscle hypoxia and increased risk of muscle tears from active or passive mobilization [7]. Anti-NMDA receptor encephalitis patients usually present with a couple of symptoms including psychiatric disturbances, memory deficits, seizures and autonomic abnormalities according to the time sequence [9]. In our patient, severe neurological symptoms, hypoxia and mechanical ventilation, epileptic seizures and delayed rehabilitation were all the risk factors for HO. Our case study suggests that patients with anti-NMDA receptor encephalitis or other autoimmune encephalitis should be closely evaluated for the chance to develop HO. In our case, the patient’s images of pelvic CT and MRI were very impressive which showed the dynamic changing process of HO. Thus it is helpful to observe these signs in the images.

Different from most HO localization after encephalitis which appears around hips, our patient had diffused ossifications in bilateral posas major and obturator internes detected by MRI scan. Since we did not find abnormal joint problem when we performed physical examination, we did not arrange X-ray examination for her. We confirmed there was no problem with her hips or joints when she revisiting 2 months later after discharging.

There was no specific laboratory examination in this case. Recent studies revealed no statistically significant difference of serum calcium and phosphorus levels between patients with head trauma and patients without head trauma [10]. PTH as a regulator of homeostasis of calcium and phosphate is considered to play an important role in enhancing bone formation. Our patient had decreased PTH. However, a recent study showed there was no significant difference of serum PTH in traumatic patients [11]. CSF tests may be normal or reveal inflammatory abnormalities [5]. Our case indicated normal CSF result.

Management includes surgical excisions, pharmacological interventions and physiotherapy for the patients who have already developed clinically significant HO. In this case, continuous passive motion was exercised under the help of family members, and the patient had recovered very well without significant deficits on movement.

Limitations on our follow-up studies include lack of images of the pelvic MRI or CT of the patient and the serum PTH result due to the financial situation of the family.

Anti-NMDA receptor encephalitis as a newly recognized autoimmune mediated disease often presents with severe neurologic symptoms and requires long time intensive care and mechanical ventilation, which makes the patient easily complicate with HO. More studies are required to elucidate the mechanisms of HO and more attentions should be paid to patients with encephalitis who might have severe muscle ossifications requiring early interventions.

## Abbreviations

Anti-NMDA: Anti-N-Methyl-D-Aspartate
CSF: Cerebral spinal fluid
CT: Computed tomography
EEG: Electroencephalograph
HO: Heterotopic ossification
ICU: Intensive care unit
IVIg: Immunoglobin
MRI: Magnetic resonance imaging
